# Susceptibility to Ebbinghaus and Müller-Lyer illusions in autistic children: a comparison of three different methods

**DOI:** 10.1186/s13229-017-0127-y

**Published:** 2017-03-23

**Authors:** Catherine Manning, Michael J. Morgan, Craig T. W. Allen, Elizabeth Pellicano

**Affiliations:** 1 Department of Experimental Psychology, University of Oxford, 9 South Parks Road, Oxford, OX1 3UD UK; 2 Applied Vision Research Centre, City University, Northampton Square, London, EC1V 0HB UK; 3 Max-Planck Institute for Metabolism Research, Gleueler Str. 50, 50931 Köln, Germany; 4 Centre for Research in Autism and Education (CRAE), UCL Institute of Education, University College London, 55-59 Gordon Square, London, WC1H 0NU UK; 5 School of Psychology, University of Western Australia, 35 Stirling Highway, Perth, WA 6009 Australia

**Keywords:** Autism, Vision, Visual illusions, Perception, Cognitive bias, Response bias, Global processing, Context

## Abstract

**Background:**

Studies reporting altered susceptibility to visual illusions in autistic individuals compared to that typically developing individuals have been taken to reflect differences in perception (e.g. reduced global processing), but could instead reflect differences in higher-level decision-making strategies.

**Methods:**

We measured susceptibility to two contextual illusions (Ebbinghaus, Müller-Lyer) in autistic children aged 6–14 years and typically developing children matched in age and non-verbal ability using three methods. In experiment 1, we used a new two-alternative-forced-choice method with a roving pedestal designed to minimise cognitive biases. Here, children judged which of two comparison stimuli was most similar in size to a reference stimulus. In experiments 2 and 3, we used methods previously used with autistic populations. In experiment 2, children judged whether stimuli were the ‘same’ or ‘different’, and in experiment 3, we used a method-of-adjustment task.

**Results:**

Across all tasks, autistic children were equally susceptible to the Ebbinghaus illusion as typically developing children. Autistic children showed a *heightened* susceptibility to the Müller-Lyer illusion, but only in the method-of-adjustment task. This result may reflect differences in decisional criteria.

**Conclusions:**

Our results are inconsistent with theories proposing reduced contextual integration in autism and suggest that previous reports of altered susceptibility to illusions may arise from differences in decision-making, rather than differences in perception per se. Our findings help to elucidate the underlying reasons for atypical responses to perceptual illusions in autism and call for the use of methods that reduce cognitive bias when measuring illusion susceptibility.

**Electronic supplementary material:**

The online version of this article (doi:10.1186/s13229-017-0127-y) contains supplementary material, which is available to authorized users.

## Background

Along with impaired social communication and interaction, autism is characterised by restricted, repetitive patterns of behaviour and interests, including atypical responses to sensory information (Diagnostic and Statistical Manual of Mental Disorders, 5th edition (DSM-5) [1]). Sensory symptoms are common in autistic individuals [2] and impact many aspects of everyday functioning, including behaviour in educational settings [3], daily living skills [4] and family life [5]. Understanding atypical sensory functioning in autism is therefore of critical import.

Vision is perhaps the sensory modality that has been most extensively studied in autistic individuals (see [6] for review). The first study to use visual illusions to characterise autistic perception was conducted by Happé [7]. She selected six illusions purported to result from the integration of features with their surrounding context: the Ponzo illusion, Ebbinghaus illusion (or Titchener’s circles), Kanisza triangle, Müller-Lyer illusion, Hering illusion and Poggendorff illusion (see Table 1). The illusions were displayed on cards, and participants were asked to make simple judgments for each illusion (e.g. Müller-Lyer: ‘are the lines the same size or different sizes?’; Ebbinghaus: ‘are the circles the same size or different sizes?’). In the size illusions (Ebbinghaus, Müller-Lyer, Ponzo), the sizes of the features were identical, so a ‘different’ judgment was deemed to result from the inducing context. Strikingly, Happé reported that young people on the autism spectrum (aged 8–16 years, *n* = 25) were susceptible to fewer visual illusions than typically developing children matched for mental age (*n* = 21) and children with a learning difficulty matched for both mental age and chronological age (*n* = 21). A smaller proportion of autistic participants succumbed to each illusion compared to the other groups, apart from in the case of the Müller-Lyer illusion, in which case the majority of autistic individuals were also ‘fooled’ by the illusion.

**Table 1:** Summary of previous studies assessing susceptibility to visual illusions in autistic individuals

Illusion and example	Study	Method	Summary of group differences in susceptibility
Ebbinghaus (or Titchener circles)	Happé [7]	Same/different	AUT < CON^a,b^
	Ropar and Mitchell [10]	Method-of-adjustment	AUT = CON
	Ropar and Mitchell [10]	Same/different	AUT = CON
	Ropar and Mitchell [12]	Method-of-adjustment	AUT ≈ CON^c^
	Schwarzkopf et al. [16]	Forced choice	AUT = CON
Müller-Lyer	Happé [7]	Same/different	AUT = CON^a,b^
	Ropar and Mitchell [10]	Method-of-adjustment	AUT > CON
	Ropar and Mitchell [10]	Same/different	AUT = CON
	Ropar and Mitchell [12]	Method-of-adjustment	AUT = CON
	Ishida et al. [13]	Method-of-adjustment	AUT = CON
Ponzo	Happé [7]	Same/different	AUT < CON^a,b^
	Ropar and Mitchell [10]	Method-of-adjustment	AUT = CON
	Ropar and Mitchell [10]	Same/different	AUT = CON
	Ropar and Mitchell [12]	Method-of-adjustment	AUT = CON
	Ishida et al. [13]	Method-of-adjustment	AUT < CON
Illusory (Kanisza) figures	Happé [7]	“How many triangles?”	AUT < CON^a^
	Milne and Scope [15]	Forced choice	AUT = CON
Poggendorff	Happé [7]	“Which line joins up with which?”	AUT < CON^a,b^
Hering	Happé [7]	“Are lines straight or curvy?”	AUT < CON^a,b^
Horizontal-vertical (or Hat)	Ropar and Mitchell [10]	Method-of-adjustment	AUT < CON
	Ropar and Mitchell [10]	Same/different	AUT = CON
	Ropar and Mitchell [12]	Method-of-adjustment	AUT < CON
Shepard’s tables	Mitchell et al. [14]	Method-of-adjustment	AUT < CON

*AUT* autism group, *CON* control group

^a^Illusion used by Hoy, Hatton and Hare [8] but individual results for each illusion not reported

^b^Illusion used by Bölte et al. [9] but individual results for each illusion not reported

^c^Individuals with Asperger’s syndrome and typically developing children aged 11 were less susceptible to the illusion than those with autism, typically developing children aged 8 and children with moderate learning difficulties

Attempts to reproduce Happé’s [7] findings have had mixed success. Hoy, Hatton and Hare [8] presented the same task used by Happé to younger autistic children, aged 4–9 years (*n* = 17), and typically developing children matched in age and verbal ability (*n* = 17), and found no group differences in the number of illusions that children with and without autism were susceptible to. Yet, Bölte, Holtmann, Poustka, Scheurich and Schmidt [9] reported reduced susceptibility to illusions in autistic adults (*n* = 15) compared to that in typical adults matched in non-verbal and verbal ability (*n* = 15) using five variants of each of five illusions (Ebbinghaus, Ponzo, Müller-Lyer, Poggendorff, Hering) in a task very similar to that used by Happé.

Other studies have used the method-of-adjustment, in which participants are asked to manipulate one stimulus until it is perceptually identical to another stimulus. In this task, participants do not need to give a verbal response, and there is scope to assess the *strength* of an illusory effect, rather than classifying responses as those that either do or do not indicate susceptibility to an illusion (cf. Happé [7]). Using this method, Ropar and Mitchell [10] found that autistic children aged 7 to 18 years (*n* = 23) were just as affected by the Ponzo and Ebbinghaus illusions as those from a range of comparison groups, including individuals with moderate learning difficulties and typically developing children and adults. Interestingly, however, the autistic children did not succumb to the horizontal-vertical illusion [11] and surprisingly showed *heightened* susceptibility to the Müller-Lyer illusion. Ropar and Mitchell further showed that there was no evidence of group differences in susceptibility to visual illusions when using a task modelled on that of Happé [7]. Ropar and Mitchell largely replicated their findings in a later study [12], demonstrating again that autistic children did not generally demonstrate a reduced susceptibility to illusions, apart from in the case of the horizontal-vertical illusion. More recently, Ishida, Kamio and Nakamizo [13] demonstrated that young people on the autism spectrum aged 10 to 16 years (*n* = 9) were less susceptible to the Ponzo illusion than typically developing children matched in age and IQ (*n* = 9) but were equally susceptible to the Müller-Lyer illusion. Furthermore, Mitchell, Mottron, Soulières and Ropar [14] presented the Shepard’s table illusion to young autistic people aged between 12 and 29 years (*n* = 18) and age- and ability-matched typically developing participants (*n* = 18) and reported that while autistic individuals were susceptible to the illusion, the illusory effect was weaker than in the comparison group.

Finally, some studies have used forced-choice methods to measure susceptibility to visual illusions in autism. Milne and Scope [15] assessed susceptibility to illusory ‘Kanisza’ figures by asking participants to judge whether a rectangle induced by surrounding shapes was ‘thin’ or ‘fat’. In this task, autistic children aged 7 to 13 years (*n* = 18) showed no differences in accuracy or reaction time compared to non-autistic children with special educational needs (*n* = 16) and typically developing children (*n* = 20). More recently, Schwarzkopf, Anderson, de Haas, White and Rees [16] used a forced-choice task using the Ebbinghaus illusion, where participants were asked to judge which stimulus was larger on each trial. In this task, adults with Asperger’s syndrome (*n* = 15) were equally susceptible to the illusion as neurotypical adults matched in age and ability (*n* = 12).

Our review of previous studies investigating visual illusions in autism presents a complex picture (see Table 1 for summary, and [17] for meta-analysis). The same is also true for studies assessing the relationship between autistic traits and illusory perception in the general population. Walter, Dassonville and Bochsler [18] reported that individuals with higher scores on the Systemizing Quotient [19] were less susceptible to some illusions (the rod-and-frame, Roelofs, Ponzo and Poggendorff illusions), but not others (induced motion, Zollner, Ebbinghaus and Müller-Lyer). Meanwhile, susceptibility to illusions was not correlated with either scores on the empathizing quotient [20] or the autism spectrum quotient (AQ) [21]. Yet, Chouinard, Noulty, Sperandio and Landry [22] later reported that higher scores on the AQ were related to reduced susceptibility to the Müller-Lyer illusion but not the Ebbinghaus and Ponzo illusions. Chouinard, Unwin, Landry and Speriando [23] later failed to replicate this result, instead showing that only the Shepard’s table and square-diamond illusions were correlated with AQ scores, out of 13 illusions tested.

While it is clear that the evidence is mixed, those studies finding group differences between autistic individuals and comparison groups have nevertheless been suggested to reflect differences in autistic perception. For example, Happé [7] proposed that autistic individuals demonstrated reduced contextual integration, processing features more independently from their surrounding context than neurotypical individuals. This explanation was tightly linked to the weak central coherence account of autism [24, 25]. It has also been suggested that reduced susceptibility to some illusions may arise from weaker top-down influences on autistic perception [14, 26]. These ideas were later elaborated in a theory of autistic perception situated within a Bayesian framework [27]. Pellicano and Burr [27] proposed that autistic individuals have attenuated (broader) priors, which means that their perception is more influenced by incoming sensory information, and is thus more veridical. Yet, some illusions may lend themselves to Bayesian explanations more easily than others [28]. For example, it is easy to postulate a role for priors in the perception of the Kanisza triangle and the hollow-face illusion, whereas illusions arising from low-level sensory processing (e.g. the Ebbinghaus illusion) may be unrelated to Bayesian inference.

It is important to consider, however, whether reports of reduced susceptibility to illusions in autism are really due to differences in perception at all. All previous studies assessing visual illusions in autism have confounded the observer’s sensitivity to an illusion with the observer’s subjective criterion for reporting whether the illusion was seen [29, 30]. Therefore, group differences in responses to illusions may have arisen due to differences in subjective criteria—or decisional bias, without necessitating underlying differences in perception: a possibility that is particularly likely when groups may differ according to cognitive and affective factors [30].^1^ Indeed, the problem of distinguishing a perceptual from a cognitive bias is not confined to studies of autism, but applies to all Type 2 psychophysical measures of bias [29] such as visual after-effects [31, 32].

**Fig. 1:**
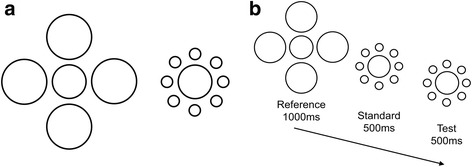
Methods for assessing the Ebbinghaus illusion. **a** Traditional method, where participants are asked whether the two stimuli have central circles that are the same size or not, and/or to judge which stimulus has the largest central circle. In this example, the central circles are identical in size. **b** Two-alternative-forced-choice method as described by Morgan et al. [29]. Participants are asked which of two sequentially presented comparison stimuli (the standard or test) has a central circle that is most similar in size to that presented in the reference. In this example, the central circle in the standard is 5% smaller than in the reference and the test is 4% larger than the standard

To circumvent this potential problem, Morgan et al. [29] advocated the use of a two-alternative forced-choice (2-AFC) procedure with a roving pedestal. Morgan et al. demonstrated how this method could be applied to a range of different perceptual phenomena. In the case of the Ebbinghaus illusion, for example, previous studies have asked autistic and non-autistic participants to determine which of two central circles is bigger (Fig. 1a). While a bias in responses could arise at the level of the percept, it could also reflect the observer’s decisional criterion (e.g. to respond that the circle surrounded by large circles is smaller when the observer is unsure). Such a criterion could be particularly affected by an observer’s previous exposure to an illusion. In Morgan et al.’s method, one reference stimulus of fixed size and two comparison stimuli are presented sequentially (Fig. 1b). One comparison stimulus (the standard) is a pedestal, which has a central circle that is either larger or smaller than that of the reference stimulus on a given trial. The other comparison stimulus (the test) has a central circle that is an increment larger than the pedestal. The two comparison stimuli have the same surrounding context circles, which differ from the context of the reference. The observer is asked whether the central circle of the first or second comparison is most similar in size to that of the reference. The order of presentation of the standard and test is randomised and the size of the pedestal (i.e. larger or smaller than the reference) is randomly interleaved throughout the task. Thus, in this task, participants cannot rely on strategies such as choosing the standard if they are unsure (as they do not know which stimulus is the standard on a given trial) or choosing a stimulus with a certain context (because they are required to choose between two stimuli with identical contexts). Using this method in conjunction with a signal detection theory framework [33], it is possible to characterise the observer’s discrimination sensitivity and the observer’s perceptual bias, whilst minimising the influence of decisional biases. Within this framework, an observer’s discrimination sensitivity is limited by ‘internal noise’ [29], which refers to any source of variability that limits performance. Differences in perceptual bias between conditions of a task (e.g. small or large context circles in the Ebbinghaus stimulus) reflect illusion susceptibility.

Given this recent methodological advance in measuring illusion susceptibility, it seems timely to revisit the question of whether autistic individuals show reduced susceptibility to illusions. In this study, we measured susceptibility to two well-characterised contextual illusions: the Ebbinghaus illusion and the Müller-Lyer illusion, in autistic and typically developing children. In experiment 1, we used Morgan et al.’s [29] method to minimise the effects of higher-level decision-making strategies, in order to measure perceptual biases as purely as possible. To allow comparison with previous studies, we used more conventional methods in experiments 2 and 3. Experiment 2 used a similar task to that used by Happé [7] and experiment 3 used a method-of-adjustment task comparable to that used by Ropar and Mitchell [12].

The Ebbinghaus and Müller-Lyer illusions are two of the most frequently used illusions with autistic populations to date (see Table 1) and have led to mixed results. Our study allowed us to investigate whether such mixed results could be attributable to methodological differences. The use of these illusions in conjunction was also informative because they are both size illusions arising from the surrounding context. Reduced contextual integration could in theory lead to reduced susceptibility for both illusions, as has been shown in the case of the Ebbinghaus illusion [7]. Yet, a distinction can be drawn between the two illusions. In the Müller-Lyer illusion, the inducing context (i.e. the fins) touches the stimulus on which judgments are made—which is not the case with the Ebbinghaus illusion. This difference may mean that the context has a greater or more automatic influence on perception for the Müller-Lyer illusion, making autistic children more susceptible to this illusion in particular [7]. The use of these illusions together therefore allows us to characterise the nature of atypical integration of context in autistic individuals. Critically, in this experiment, we examine whether any differences in illusory perception between autistic children and typically developing children can be revealed when using rigorous methods to minimise the influence of cognitive bias.

## Methods

### General procedure

This study measured susceptibility to Ebbinghaus and Müller-Lyer illusions in autistic and typically developing children using three methods: one specifically designed to minimise cognitive biases (experiment 1) and two to allow comparison with previous studies (experiments 2 and 3). Children were tested individually in a quiet room as part of a wider battery of tasks within sessions of a public engagement of science event. Computer tasks were presented with a viewing distance of 50 cm. When children completed more than one experiment, the experiments were presented sequentially (i.e. experiment 1 was followed by experiment 2 and then experiment 3).

### Participants

Autistic and typically developing children aged 6 to 14 years were recruited from schools and community contacts in the Greater London area. Autistic children had previously received an independent clinical diagnosis of an autism spectrum condition according to International Classification of Diseases (ICD-10) [34] or DSM-IV [35] criteria. Typically developing children had no diagnosed developmental conditions, as reported by parents. Parents completed the Social Communication Questionnaire (SCQ) [36], and autistic children were administered the Autism Diagnostic Observation Schedule-2nd edition (ADOS-2) [37]. All autistic children scored above threshold for an autism spectrum condition on one or both measures, and no typically developing child scored above the threshold on the SCQ (score of 15; [36]). All children were cognitively able (IQ > 70), as assessed by the Wechsler Abbreviated Scales of Intelligence, Second Edition (WASI-II) [38]. Further details on the participants included in each experiment are provided below.

### Apparatus and stimuli

Computer tasks (experiments 1 and 3) were presented on a Dell Precision laptop (1366 × 768 pixels, 60 Hz) using MATLAB and elements of the Psychophysics Toolbox [39–41]. White stimuli were presented on a mid-grey background, at 61% Weber contrast.

## Experiment 1: 2-AFC roving pedestal

### Participants

The dataset for the Ebbinghaus analysis included 29 autistic children (4 females) and 33 typically developing children (12 females). The groups did not differ significantly in age, *t*(60) = 1.49, *p* = .14, or non-verbal ability, *t*(60) = .41, *p* = .69, although the autistic children had lower verbal IQ scores, *t*(40.53) = 2.93, *p* = .006 (see Table 2 for scores). The dataset for the Müller-Lyer analysis included 33 autistic children (4 females) and 47 typically developing children (18 females). The groups did not differ significantly in age, *t*(78) = 1.36, *p* = .18, or non-verbal ability, *t*(78) = .33, *p* = .74, but differed in verbal ability, *t*(78) = 4.55, *p* < .001 (see Table 2 for scores). Twenty-one autistic children and 11 typically developing children were in the datasets for both the Ebbinghaus and Müller-Lyer versions of the experiment.

**Table 2:** Characteristics of participants for each task in experiment 1

	Ebbinghaus		Müller-Lyer	
Characteristic	Autistic	Typically developing	Autistic	Typically developing
N	29	33	33	47
Age	10.09 (2.06)	9.33 (1.95)	10.46 (2.00)	9.86 (1.95)
	6.27–14.28	6.63–13.47	7.41–14.28	6.18–13.75
PIQ	101.66 (16.70)	103.27 (14.70)	100.48 (14.28)	101.53 (13.29)
	75–141	78–131	75–128	74–131
VIQ	98.28 (17.22)	108.67 (8.82)	97.48 (14.22)	110.60 (11.49)
	71–130	91–132	73–126	77–135
FSIQ	99.69 (16.02)	106.85 (10.96)	98.67 (12.59)	107.13 (11.39)
	73–129	90–131	76–119	81–131
SCQ	22.62 (6.57)	4.63 (3.19)	22.55 (6.68)	3.90 (3.25)
	5–33	0–12	5–35	0–13
ADOS total	11.37 (5.49)		10.60 (4.83)	
	2–22		2–22	

Mean (SD) range. IQ scores were assessed using the Wechsler Abbreviated Scales of Intelligence (WASI-II) [38]

*SCQ* Social Communication Questionnaire [36], *ADOS* Autism Diagnostic Observation Schedule-2 [37], *VIQ* verbal IQ, *PIQ* performance IQ, *FSIQ* full-scale IQ

An additional five autistic children and two typically developing children were excluded from the Ebbinghaus analysis, and an additional four autistic children and one typically developing child were excluded from the Müller-Lyer analysis due to poor-fitting psychometric functions (see “Data screening and analysis” section). A further four of the youngest typically developing children were removed from each of the Ebbinghaus and Müller-Lyer datasets to ensure the groups matched adequately in age.

### Stimuli

**Fig. 2:**
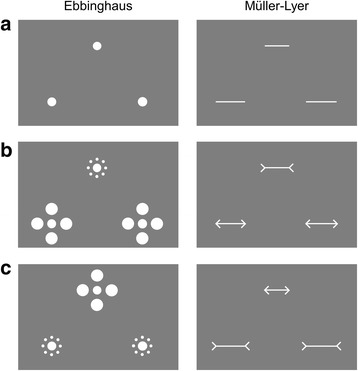
Schematic representation of stimuli used in experiment 1. **a** Context-free practice trial. **b** S-L context condition in Ebbinghaus task and O-I context condition in Müller-Lyer task. **c** L-S context condition in Ebbinghaus task and I-O in Müller-Lyer task. In all examples, the reference stimulus is presented at the top and the two comparison stimuli are presented below. In these examples, the left comparison stimulus is the standard (pedestal) and the right comparison stimulus is the test

The reference stimulus was centred horizontally, at the top of the screen. The comparison stimuli were positioned below the reference stimulus, to form a triad (see Fig. 2). In the Ebbinghaus task, the diameter of the central circle of the reference stimulus was fixed at 1.25°. The stimuli were either surrounded by eight small context circles with a diameter of .42° and positioned 1.25° from the centre of the stimulus or by four large context circles with a diameter of 1.67°, positioned 2.08° from the centre of the stimulus. In the Müller-Lyer task, the reference stimulus had a horizontal line that was 3° in length. Fins were 1° long, attached to the end of the horizontal line at an angle of 45° (either inward or outward).

### Procedure

The task was based on the Ebbinghaus task devised by Morgan et al. [29], with three main modifications to make it suitable for child participants. First, the task was presented within the context of a developmentally appropriate trading game. Second, to minimise memory demands, the reference stimulus was always present on the screen, and the comparison stimuli were presented simultaneously (cf. [29]). Third, to reduce the number of trials, we omitted the context-free condition.

The reference stimulus was presented continuously on the screen. The experimenter initiated each trial with a keypress, triggering the presentation of comparison stimuli for a duration of 1000 ms. In the Ebbinghaus version of the experiment, children were asked to identify which of the two comparison stimuli had a central circle most similar in size to that of the reference stimulus. In the Müller-Lyer version of the experiment, children were asked to identify which of the two comparison stimuli had a horizontal line most similar in size to that of the reference stimulus. Before completing the experimental trials, the task was explained to participants with four context-free demonstration trials (Fig. 2a) and four demonstration trials with context. Participants were also shown examples of stimuli on cards where necessary.

Children were told that they were trading shapes in ‘The Bank of Geometrica’. The reference stimulus was the ‘most valuable shape’ in the game, and participants had to choose which of the two comparison stimuli was the most similar in size to this stimulus. Participants were told that they would be able to trade the shapes that they had chosen for points. Throughout the session, children made their responses verbally (left/right) or by pointing, and the experimenter entered their responses using a keyboard. No specific feedback on performance was given although general encouragement was provided throughout.

The participants completed the task in two context conditions, which were presented sequentially in a counterbalanced order. In one condition of the Ebbinghaus task (S-L; Fig. 2b), the reference stimulus had small context circles and the comparison stimuli had large context circles; in the other condition (L-S; Fig. 2c), the reference stimulus had large context circles and the comparison stimuli had small context circles. In the Müller-Lyer task, one condition (O-I; Fig. 2b) had outward fins on the reference and inward fins on the comparison stimuli, and the other condition (I-O; Fig. 2c) had inward fins on the reference and outward fins on the comparison stimuli. One comparison stimulus was a standard, and the other comparison stimulus was a test.

For each context condition, participants completed 40 trials in which the standard was a pedestal below the reference, and 40 trials in which the standard was a pedestal above the reference. In the Ebbinghaus task, the central circle of the standard was either −5 or +5% of the diameter of that in the reference stimulus (i.e. 1.19° or 1.31°). In the Müller-Lyer task, the length of the horizontal line in the standard was either −20 or +20% of the length of that in the reference stimulus (i.e. 2.4° or 3.6°).^2^ The pedestals were randomly interleaved throughout the task (i.e. a ‘roving pedestal’; [29, 42]). The location of the standard stimulus (left or right) was randomised on each trial. The size of the test stimulus was guided by method of constant stimuli, with eight trials presented at five different levels for each pedestal (+1, +2, +4, +8, +16% of the diameter or length of the standard for the Ebbinghaus and Müller-Lyer experiments, respectively). These trials were presented in a randomised order.

The 80 trials for each context condition were divided into blocks of 20 trials. After each block, participants were shown a cartoon cash register which calculated the ‘points’ they had obtained. These points were randomly allocated but provided motivation for children throughout the task. Each context condition took approximately 5 min.

### Data screening and analysis

The psychophysical task is a comparison-of-comparisons task [42]. Using a signal detection theory approach [33], the standard (*S*) and test (*T*) stimuli can each be described by normal distributions with mean values corresponding to the physical size of the stimulus (*p, p + t*) plus perceptual bias (*μ*) and variances (*σ* ^*2*^) corresponding to performance-limiting internal noise [42]:

$$ \begin{array}{c}\hfill \mathrm{S} \sim N\left( p+\mu,\ {\sigma}^2/2\right)\hfill \\ {}\hfill \mathrm{T} \sim N\left( p+ t + \mu,\ {\sigma}^2/2\right)\hfill \end{array} $$

Thus, the probability of choosing the standard can be calculated as:

$$ \begin{array}{l}\mathrm{P}(S) = \mathrm{P}\left(\left| S\right| < \left| T\right|\right)\\ {} = \mathrm{P}\left({S}^2/{T}^2 < 1\right)\end{array} $$

where *S* ^2^/*T* ^2^ is a random variable with a doubly non-central *F* distribution [42].

**Fig. 3:**
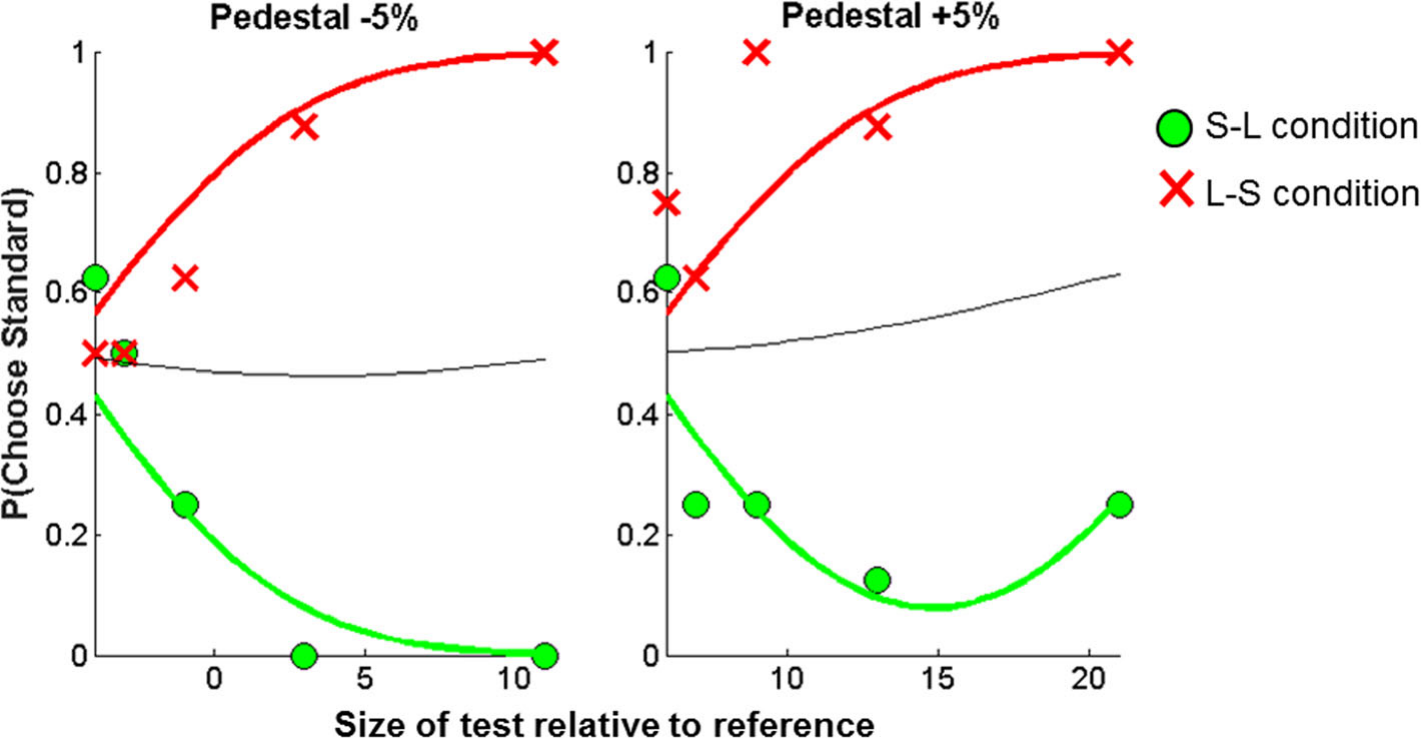
Example dataset for the Ebbinghaus task. Maximum likelihood fits to the data for an 11-year-old typically developing child in the Ebbinghaus task, including two pedestal values (−5, +5%) and two context conditions (S-L: where the reference stimulus has small context circles and the comparison stimuli have large context circles, and L-S: where the reference has large context circles and the comparison stimuli have small context circles). The *red* and *green lines* represent fits to the data where internal noise (*σ*) is constant across context conditions, but bias (*μ*) is free to vary. The *black line* represents the fit of a single model where bias and internal noise are both held constant across the context conditions

Maximum likelihood psychometric functions were fit to each participant’s data, for each combination of pedestal and context condition, assuming constant internal noise across conditions, but allowing bias to vary across the context conditions. Figure 3 shows psychometric functions for a typically developing child. In the S-L condition, the central circle of the reference stimulus tends to appear bigger than the central circles of the comparison stimuli. Thus, as the test is made larger than the pedestal in the negative pedestal trials (−5%), the participant becomes less likely to choose the standard (or pedestal), as s/he perceives the larger comparison stimulus (i.e. the test) to be most similar in size to the reference stimulus. In the positive pedestal condition, the participant may become more likely to choose the test as it increases in size, until it exceeds a limit at which the pedestal starts to look more similar in size to the reference stimulus. In the L-S condition, the central circle of the reference stimulus tends to appear smaller than the central circles of the comparison stimuli. Thus, as the test is made larger than the standard, the participant becomes more likely to choose the standard as it is the smallest stimulus. Thus, a negative bias is expected in condition S-L, and a positive bias is expected in condition L-S. The same logic can be applied to the Müller-Lyer illusion, whereby a negative bias is expected in condition O-I and a positive bias in condition I-O.

Assuming that the different context conditions are associated with the same value of internal noise, but different values of bias, we obtained one internal noise (*σ*) and two bias (*μ*) parameters, taking into account the pedestal value and fitted context bias for each observation [29, 42]. Internal noise and bias are expressed as Weber fractions with respect to the size of the reference stimulus (%).

We screened the data for poorly fitting psychometric functions and removed datasets where the likelihood of the fit was particularly low (log likelihood <−110) and/or the internal noise value was 30 or above such as to make the slope of the psychometric function essentially flat. Such functions suggested that participants were not successfully discriminating between stimuli, which may have been due to inattentiveness or a lack of task understanding. Five autistic children and two typically developing children were removed from the Ebbinghaus analysis, and four autistic children and one typically developing child were removed from the Müller-Lyer analysis on this basis.

To quantify the extent of bias associated with each illusion, we calculated the difference in bias between the two context conditions (i.e. Bias_L-S_ − Bias_S-L_ or Bias_I-O_ − Bias_O-I_ for the Ebbinghaus and Müller-Lyer tasks, respectively). Internal noise values were log-transformed to minimise the effects of skewness and kurtosis. Outliers—defined as points lying 3 or more standard deviations from the group mean—were replaced with points lying 2.5 standard deviations from the group mean [43]. Two outliers were identified in the bias values for the Ebbinghaus task (autistic *n* = 1; typically developing *n* = 1), and a further two were identified in the bias values for the Müller-Lyer task (autistic *n* = 1; typically developing *n* = 1), which were replaced with trimmed values. Note that the same pattern of results was obtained without outlier replacement (see Additional file 1). Shapiro-Wilks tests showed that the distribution of log-transformed internal noise values did not differ significantly from a normal distribution, in either task (*p*s ≥ .53). However, the bias values deviated from normality in both tasks (*p*s < .001). We therefore supplemented our analyses on these variables with bootstrapped analyses with 1000 samples, and bias-corrected 95% confidence intervals to ensure our results were robust to deviations from normality.

## Results and discussion

**Fig. 4:**
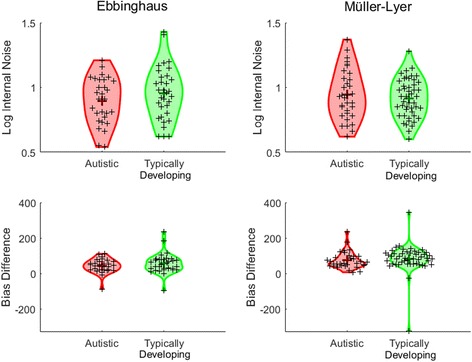
Internal noise and bias estimates for autistic children and typically developing children in experiment 1. Individual data points (*small crosses*) and group means (*large crosses*) are shown for Ebbinghaus stimuli (*left panel*) and Müller-Lyer stimuli (*right panel*). Distributions smoothed with kernel density functions are shown in *red* (autistic children) and *green* (typically developing children). Data are presented with outliers trimmed

The values of internal noise and perceptual bias in autistic and typically developing children are shown in Fig. 4. The average bias associated with the Müller-Lyer illusion (autistic: *M* = 73.97, SD = 47.51; typically developing: *M* = 83.51, SD = 79.79) was greater than that associated with the Ebbinghaus illusion (autistic: *M* = 42.63, SD = 39.07; typically developing: *M* = 55.84, SD = 55.91), although there was considerable individual variability. There was no significant difference in the extent of bias displayed by autistic children and typically developing children in the Ebbinghaus task, *t*(60) = 1.06, *p* = .29 (bootstrapped 95% CI for mean difference: [−38.11, 9.61]; *p* = .29), and no significant group difference in internal noise estimates, *t*(60) = 1.17, *p* = .25 (autistic: *M* = .90, SD = .18; typically developing: *M* = .95, SD = .21). Likewise, the groups did not differ significantly in terms of bias, *t*(78) = .61, *p* = .54 (bootstrapped 95% CI for mean difference: [−38.17, 19.42]; *p* = .52), or internal noise, *t*(78) = .78, *p* = .44 (autistic: *M* = .94, SD = .19; typically developing: *M* = .91, SD = .15), in the Müller-Lyer experiment.

We conducted correlational analyses to investigate whether participant characteristics contributed to differences between participants. Internal noise in the Ebbinghaus experiment was negatively related to age [*r* = −.40, *p* = .001], with older children having lower levels of internal noise. Internal noise in the Müller-Lyer experiment was negatively related to both verbal IQ [*r* = −.27, *p* = .02] and non-verbal IQ [*r* = −.24, *p* = .03], with higher internal noise values associated with lower ability. To ensure that group differences in verbal IQ were not contributing to the results, we confirmed that there was no significant group difference in internal noise in the Müller-Lyer task whilst covarying the effect of verbal ability, *F*(1,77) = .12, *p* = .73. All other correlations between task measures and age and ability were non-significant, *p*s ≥ .11.

To ensure that the non-significant difference in bias between autistic and typically developing children could not be accounted for by data insensitivity [44, 45], we quantified the relative evidence for the null and alternative hypotheses using the Bayesian independent *t* tests with a default Cauchy prior width of 1, implemented using JASP software [46]. The Bayes factors (BF) resulting from these tests reflect a continuum of evidence favouring the null and alternative hypotheses, with BF < 1/3 providing substantial evidence for the null hypothesis and BF > 3 providing substantial evidence for the alternative hypothesis [47]. The results confirmed that there was substantial evidence in support of the null hypothesis of no group differences in bias in both the Ebbinghaus (BF = .32) and Müller-Lyer (BF = .21) experiments. Robustness checks assessing the influence of the choice of prior are provided in Additional file 2.

These results show that autistic children do not show altered susceptibility to the Ebbinghaus and Müller-Lyer illusions when using a novel method that minimises decision bias. For comparison, we next measured susceptibility to the same illusions using more traditional methods. In experiment 2, we revisited the paradigm used by Happé [7].

## Experiment 2: same-different responses

### Participants

In the Ebbinghaus task, the dataset included 21 children in the autistic group (2 females) and 28 children in the typically developing group (11 females), with no differences between the groups in terms of age, *t*(47) = .93, *p* = .36, non-verbal ability, *t*(31.38) = 1.37, *p* = .18, or verbal ability, *t*(47) = 1.75, *p* = .09 (see Table 3 for scores). In the dataset for the Müller-Lyer task, there were 24 autistic children (1 female) and 42 typically developing children (15 females), matched in age, *t*(64) = 1.20, *p* = .23, and non-verbal ability, *t*(32.23) = .23, *p* = .82, but not in verbal ability, *t*(64) = 4.01, *p* < .001 (see Table 3 for scores). Seventeen autistic children and 19 typically developing children were included in the datasets for both versions of the experiment. Twelve autistic children and four typically developing children in the Ebbinghaus dataset were also included in the Ebbinghaus dataset in experiment 1, and 17 autistic children and 26 typically developing children included in the Müller-Lyer dataset were also in the Müller-Lyer dataset in experiment 1. A further two autistic children and 14 typically developing children were excluded from analysis in the Ebbinghaus task, and an additional five autistic children and 20 typically developing children were excluded from the analysis in the Müller-Lyer task because they incorrectly responded that the control stimuli differed in size.

**Table 3:** Characteristics of participants for each task in experiment 2

	Ebbinghaus		Müller-Lyer	
Characteristic	Autistic	Typically developing	Autistic	Typically developing
N	21	28	24	42
Age	9.88 (1.97)	9.34 (2.05)	9.94 (1.96)	9.32 (2.05)
	7.38–14.73	6.09–13.86	7.38–14.73	6.09–13.86
PIQ	108.14 (20.53)	101.18 (12.80)	103.96 (20.89)	102.88 (12.25)
	75–141	75–131	75–141	75–131
VIQ	100.95 (16.87)	108.68 (14.05)	97.58 (15.04)	112.62 (14.45)
	73–130	77–130	73–126	77–149
FSIQ	105.00 (17.27)	105.96 (12.79)	100.71 (16.34)	109.07 (12.73)
	73–129	78–131	73–129	78–135
SCQ	22.32 (6.91)	3.67 (3.10)	21.68 (6.50)	3.59 (3.24)
	8–38	0–12	11–33	0–13
ADOS total	11.75 (4.62)		11.96 (4.54)	
	3–21		7–21	

Mean (SD) range. IQ scores were assessed using the Wechsler Abbreviated Scales of Intelligence (WASI-II) [38]

*SCQ* Social Communication Questionnaire [36], *ADOS* Autism Diagnostic Observation Schedule-2 [37], *VIQ* verbal IQ, *PIQ* performance IQ, *FSIQ* full-scale IQ

### Stimuli

Stimuli were presented on A4 laminated cards, with three cards for each of the Ebbinghaus and Müller-Lyer illusions. The stimuli were presented in white on a mid-grey background, as in experiment 1. For each illusion, there was a context-free condition, where two circles (diameter .9 cm) or two horizontal lines (length 2.1 cm) were presented side-by-side for the Ebbinghaus and Müller-Lyer tasks, respectively. There were also two cards for each illusion that had the same stimuli with added context. For the Ebbinghaus illusion, one card had four large context circles (diameter 1.1 cm) on the left and eight small context circles (diameter .3) on the right, and the other card had small context circles on the left and large context circles on the right. For the Müller-Lyer illusion, one card had inward fins on the left and outward fins on the right, and the other card had outward fins on the left and inward fins on the right. The fins were .7 cm in length and were oriented at 45° as in experiment 1. The central circles and horizontal lines were always the same size. The relative sizes and configurations of the context circles and fins were the same as in experiment 1.

### Procedure

The cards were shuffled to randomise the order of presentation, and the experimenter held up one card at a time. Following Happé [7], children were either asked ‘Are the [circles/lines] the same size or different sizes?’ or ‘Are the [circles/lines] different sizes or the same size?’. The question order was counterbalanced across participants. If children responded ‘different’, they were asked to identify which was bigger. Children were prevented from touching the cards while making their judgments.

### Data screening and analysis

Following Happé [7], participants were only included in the analysis if they correctly responded that the circles/lines were the same size in the context-free condition. We then counted the number of cards displaying context for which participants gave the expected incorrect judgment, yielding a score ranging from 0 to 2 for each illusion.

### Results and discussion

**Fig. 5:**
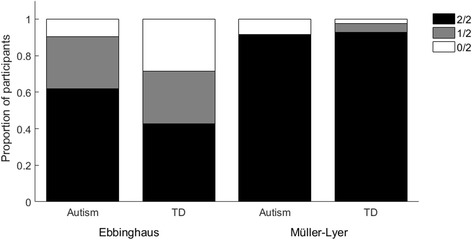
Proportions of autistic and typically developing (TD) children succumbing to the Ebbinghaus and Müller-Lyer illusions in neither, one or both trials, in experiment 2

Out of 21 autistic children, 13 (61.9%) succumbed to the Ebbinghaus illusion on both trials, 6 (28.6%) succumbed to the Ebbinghaus illusion on one trial only and 2 (9.5%) did not succumb to the illusion on either trial (Fig. 5). Out of 28 typically developing children, 12 (42.9%) succumbed to the Ebbinghaus illusion on both trials, 8 (28.6%) succumbed to the illusion on one trial only and 8 (28.6%) did not succumb to the illusion at all. Chi-squared analysis (with Yates correction) revealed no significant differences between the groups in the number of children who never succumbed to the illusion and the number of children who succumbed to the illusion in one or more trial, *χ* ^2^(1) = 1.64, *p* = .20. Logistic regression revealed that age and ability were not significant predictors of whether children succumbed to the illusion or not, *p*s ≥ .16.

In the Müller-Lyer task, 22 out of 24 autistic children (91.7%) succumbed to the illusion on both trials, while the remaining two children did not succumb to the illusion on either trial (8.3%). Thirty-nine out of 42 typically developing children (92.9%) succumbed to the illusion on both trials, 2 (4.76%) succumbed to the illusion on one trial and 1 did not succumb to the illusion on either trial (2.38%). The proportions of participants who succumbed to the illusion on one or more trials compared to those who never succumbed to the illusion did not differ between the autistic children and typically developing children, *χ* ^2^(1) = .25, *p* = .62 (with Yates correction). Age and ability did not significantly predict susceptibility in a logistic regression (*p*s ≥ .08).

Bayesian contingency tables with independent multinomial sampling and a prior concentration of 1 implemented in JASP software [46, 48] were also used to compare group differences in the number of children who succumbed to the illusion on no trials, one trial or both trials. The null hypothesis is that there is independence between groups and responses, and the alternative hypothesis is that there is an association between the groups and responses. The results revealed substantial evidence for the null hypothesis in the Müller-Lyer experiment (BF = .07) but inconclusive evidence for either the null or alternative hypothesis in the Ebbinghaus task (BF = .64). Therefore, the data were insensitive to group differences in the Ebbinghaus task and larger samples would be required to conclusively determine whether the groups differ in this task. Robustness checks showing the influence of prior concentration can be found in Additional file 2.

In sum, it appears that a similar proportion of autistic children are susceptible to the Ebbinghaus and Müller-Lyer illusions as typically developing children in this simple judgment task, although more data is required in the Ebbinghaus task. In line with the results from experiment 1, a greater proportion of children were susceptible to the Müller-Lyer illusion compared to the Ebbinghaus illusion, suggesting that it is a more compelling illusion for both autistic and typically developing children. As in experiment 1, there was no difference between groups, even for a measure which is contaminated by decision bias. However, these binary judgments (same/different) may not be sufficiently fine-grained to reveal subtle group differences. Indeed, it is possible that the majority of autistic children experience the illusions, but the strength of their effects may differ from that experienced by typically developing children. In experiment 3, we therefore used the method-of-adjustment.

## Experiment 3: method-of-adjustment

### Participants

Nineteen autistic children (1 female) and 38 typically developing children (15 females) completed the Ebbinghaus method-of-adjustment task. The groups were comparable in terms of age, *t*(55) = 1.13, *p* = .26, non-verbal ability, *t*(27.04) = 1.03, *p* = .31, and verbal ability, *t*(55) = 1.59, *p* = .12 (see Table 4 for scores). Twenty-four autistic children (1 female) and 58 typically developing children (20 females) completed the Müller-Lyer method-of-adjustment task. The groups of autistic children and typically developing children were matched in terms of age, *t*(80) = .75, *p* = .46, and non-verbal ability, *t*(30.48) = .29, *p* = .78, but the autistic children had lower verbal IQs than typically developing children, *t*(80) = 4.02, *p* < .001 (see Table 4 for scores). Seventeen of the autistic children and 36 of the typically developing children participated in both the Ebbinghaus and Müller-Lyer versions of the experiment.

**Table 4:** Characteristics of participants for each task in experiment 3

	Ebbinghaus		Müller-Lyer	
Characteristic	Autistic	Typically developing	Autistic	Typically developing
N	19	38	24	58
Age	10.06 (1.97)	9.44 (1.95)	9.93 (2.11)	9.56 (12.76)
	7.41–14.73	6.09–13.86	7.38–14.73	74–131
PIQ	106.32 (18.42)	101.47 (12.84)	103.08 (20.72)	101.78 (12.76)
	76–141	75–131	75–141	74–131
VIQ	102.21 (16.18)	108.61 (13.31)	98.38 (13.97)	111.64 (13.44)
	73–130	77–135	73–126	77–149
FSIQ	104.74 (15.74)	105.97 (11.93)	100.67 (15.97)	107.84 (12.22)
	77–129	78–131	73–129	78–135
SCQ	22.83 (6.26)	3.97 (2.99)	22.22 (7.25)	3.62 (3.22)
	13–38	0–12	11–38	0–13
ADOS total	10.56 (4.73)		11.22 (5.01)	
	3–21		3–21	

Mean (SD) range. IQ scores were assessed using the Wechsler Abbreviated Scales of Intelligence (WASI-II) [38]

*SCQ* Social Communication Questionnaire [36], *ADOS* Autism Diagnostic Observation Schedule-2 [37], *VIQ* verbal IQ, *PIQ* performance IQ, *FSIQ* full-scale IQ

In the Ebbinghaus dataset, 16 autistic children and 27 typically developing children were included in the Ebbinghaus dataset in experiment 2, and 11 autistic children and eight typically developing children were included in the Ebbinghaus dataset in experiment 1. In the Müller-Lyer dataset, 20 autistic children and 39 typically developing children were also included in the Müller-Lyer dataset in experiment 2, and 15 autistic children and 39 typically developing children were included in the Müller-Lyer dataset in experiment 1.

### Stimuli

Two stimuli were presented side-by-side on the screen, in the same configuration as in experiment 2. In the Ebbinghaus task, one stimulus had small context circles, and one stimulus had large context circles. In the Müller-Lyer experiment, one stimulus had inward fins and one stimulus had outward fins. The context locations (i.e. whether the small context circles or inward fins were on the left or right) were counterbalanced among participants. The sizes of the context circles and fins were the same as in experiment 1. One stimulus was a reference stimulus, with the same dimensions as in experiment 1. The other was a comparison, in which the initial diameter of the central circle or length of the horizontal line was randomised between .68° and 1.82° or 2.43° and 3.86°, respectively.

### Procedure

Children were asked to adjust the size of the comparison stimulus to match the size of the reference stimulus. The location of the comparison stimulus was signalled with a small green rectangle for 1000 ms before the stimuli appeared. Children used up and down arrow keys to make the comparison stimulus bigger or smaller, respectively, and pressed the space bar when they were satisfied that the two stimuli were the same size. There was no time limit. The task was presented in the context of a factory, ‘GeoFactory’, in which children were asked to make a shape that was the same as the one in the catalogue (i.e. the reference stimulus).

Children were initially presented with a practice trial with a star shape, to familiarise them with the task and the response keys. Next, eight experimental trials were presented. Four trials were context-free (i.e. without context circles or fins), and four trials had context. We counterbalanced across participants whether the context-free or context trials were presented first. The locations of the reference and comparison stimulus (left/right) were varied across trials. In the Ebbinghaus task, there were two trials where the reference stimulus was surrounded by small context circles and two trials where the reference stimulus was surrounded by large context circles, and in the Müller-Lyer task, there were two trials where the reference stimulus was flanked by outward fins and two trials where it was flanked by inward fins. We refer to these conditions as S-L and L-S and O-I and I-O, respectively, for comparison with experiment 1. The order of trials was randomised.

### Data screening and analysis

We computed the difference between the size of the adjusted comparison stimulus and the reference stimulus, as a proportion of the size of the reference stimulus, in context-free and context trials, before taking an average of the context-free trials and the trials in each context condition (S-L and L-S in the Ebbinghaus task and O-I and I-O in the Muller-Lyer task). As in experiment 1, a single value of bias was computed by calculating the difference between the two context conditions. As in experiment 1, points lying 3 or more standard deviations away from the group mean were replaced with those lying 2.5 standard deviations from the mean. There were no outliers in the Ebbinghaus task. One outlying value was found (an autistic child) for the Müller-Lyer task (context-free condition). Note that the same pattern of results was obtained when this outlying value was retained without replacement (Additional file 1).

We also recorded response time and the number of keypresses between the stimulus onset and children pressing the space bar to indicate they had completed the trial. These values were log-transformed to minimise the effects of negative skew and subjected to outlier screening, although no outliers were found. Shapiro-Wilks tests showed that the distribution of context-free size judgments in the Ebbinghaus task and the bias values in the Müller-Lyer experiment did not differ significantly from a normal distribution (*p* = .06 and *p* = .25, respectively). However, the bias values in the Ebbinghaus task and the context-free judgments in the Müller-Lyer task did deviate from normality (*p* = .007 and *p* < .001, respectively). Where the assumption of normality was violated, we conducted bootstrapped analyses as in experiment 1.

### Results and discussion

**Fig. 6:**
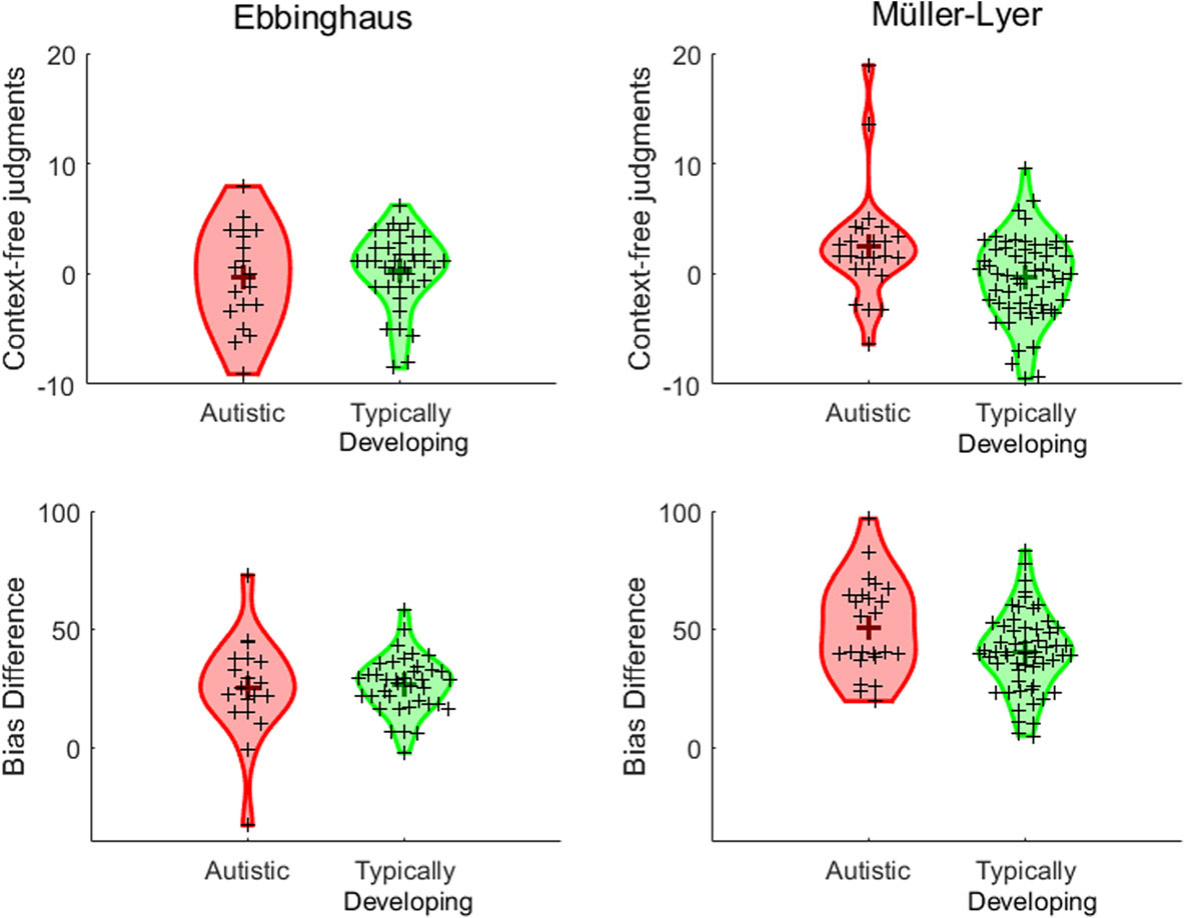
Judgments made in the context-free trials and the extent of bias in the context trials in experiment 3, for autistic and typically developing children. Individual data points (*small crosses*) and group means (*large crosses*) are shown for Ebbinghaus stimuli (*left panel*) and Müller-Lyer stimuli (*right panel*). Distributions smoothed with kernel density functions are shown in *red* (autistic children) and *green* (typically developing children). Data are presented with outliers trimmed

Individual and group results for context-free size judgments and bias estimates are shown in Fig. 6. First, we assessed group differences in the Ebbinghaus task. On average, the autistic children made the comparison stimulus slightly smaller than the reference stimulus in the context-free condition of the Ebbinghaus task, and the typically developing children made it slightly larger. However, the confidence intervals spanned 0 in both groups, suggesting that their perception was largely accurate (autistic: *M* = −.30, SD = 4.51, 95% CI = [−2.47, 1.87]; typically developing: *M* = .34, SD = 3.40, 95% CI = [−.77, 1.46]). Moreover, the groups did not differ significantly in their judgments, *t*(55) = .60, *p* = .55 (autistic: *M* = 25.29, SD = 21.24; typically developing: M = 26.53, SD = 12.08). Next, we compared the bias associated with the context in the Ebbinghaus illusion and found that the groups did not differ significantly, *t*(55) = .28, *p* = .78 (bootstrapped 95% CI for mean difference: [−12.56, 9.34], *p* = .83). Neither the bias nor the context-free size judgment was significantly related to age and verbal or non-verbal IQ, *p*s ≥ .53.

In contrast, the groups differed in their responses to context-free trials in the Müller-Lyer task, *t*(80) = 2.78, *p* = .007, *d* = .63 (bootstrapped 95% CI for mean difference: [.89, 4.99], *p* = .02). The autistic children had a tendency to make the comparison larger than the reference (*M* = 2.44, SD = 5.13, 95% CI = [.28, 4.61]), whereas the typically developing children were more accurate (*M* = −.39, SD = 3.75, 95% CI = [−1.37, .61]). There were also differences in the extent of bias in the context trials, with the autistic children showing a *larger* bias (*M* = 51.03, SD = 19.70) than typically developing children (*M* = 40.44, SD = 16.64), *t*(80) = 2.48, *p* = .015, *d* = .58. Neither the context-free judgments nor the bias values were significantly related to age and verbal or non-verbal ability (*p*s ≥ .06). As in experiment 1, the Müller-Lyer illusion was associated with a greater level of bias than the Ebbinghaus task, overall.

Next, we investigated group differences in response times and numbers of keypresses (Table 5). There were no significant group differences in response times in either the Ebbinghaus or Müller-Lyer tasks, and no interactions between group and context condition (context-free, context), *p*s ≥ .16. Thus, response times were not analysed further. We then investigated the number of keypresses. In the Ebbinghaus task, a mixed ANOVA with group as a between-participants factor and context condition as a within-participants factor showed no significant effect of group nor interaction between group and condition in the number of keypresses (*p*s ≥ .13). However, in the Müller-Lyer task, the autistic children used significantly more keypresses than typically developing children in the Müller-Lyer task, *F*(1,80) = 14.11, *p* < .001, *ɳ*_p_^2^ = .15. The effect of group did not interact with context condition (*p* = .88). In the Müller-Lyer task, the number of keypresses in the context-free condition was significantly correlated with the corresponding size judgment, *r*(80) = .32, *p* = .003, and the number of keypresses in the context condition was significantly correlated with the extent of bias, *r*(80) = .67, *p* < .001, suggesting that increased keypresses reflect size judgements in this task.

**Table 5:** Means and standard deviations of response times (RT) in seconds and number of presses before making a decision in the context-free and context trials of the Ebbinghaus and Müller-Lyer tasks in experiment 3, for autistic and typically developing children

	Ebbinghaus				Müller-Lyer			
Group	Context-free		Context		Context-free		Context	
	RT	Presses	RT	Presses	RT	Presses	RT	Presses
Autistic	.95 (.21)	1.15 (.33)	1.12 (.20)	1.34 (.43)	1.04 (.26)	1.26 (.33)	1.16 (.21)	1.60 (.19)
Typically developing	.97 (.14)	1.09 (.20)	1.08 (.15)	1.18 (.24)	1.02 (.16)	1.07 (.26)	1.16 (.18)	1.42 (.23)

Values have been log-transformed to minimise the effects of skewness and kurtosis

As in the other experiments, we complemented our analysis of differences in bias with Bayesian statistics. In line with the results of our frequentist statistics, we found substantial evidence in support of the null hypothesis of no group differences in bias in the Ebbinghaus task (BF = .22). While there was relatively more evidence in favour of the alternative hypothesis in the Müller-Lyer task (BF = 2.87), this only constituted weak/anecdotal evidence, suggesting that more data is required to provide strong evidence. Robustness checks for these Bayesian *t* tests are provided in Additional file 2.

## General discussion

In this study, we used three methods to characterise responses to Ebbinghaus and Müller-Lyer illusions in children on the autism spectrum and typically developing children. The first of these methods was designed to reduce the influence of decision biases on judgments, whereas the other two were methods that have been used previously with autistic populations and which may be contaminated by decision biases. Across all methods, the Müller-Lyer illusion had stronger effects on responses compared to the Ebbinghaus illusion. However, we were particularly interested in comparing the responses between autistic and typically developing children. We found no evidence of reduced susceptibility to the Ebbinghaus illusion in autistic children for any method. There was some indication of *heightened* susceptibility to the Müller-Lyer illusion, but only in a method-of-adjustment task (experiment 3) and not in the 2-AFC or the same-different methods (experiments 1 and 2).

The evidence we found for heightened susceptibility to the Müller-Lyer illusion in the method-of-adjustment (albeit relatively weak) was not entirely unexpected. Ropar and Mitchell [10] similarly reported a pronounced bias in response to the Müller-Lyer illusion for autistic children aged 7 to 18 years using a method-of-adjustment task. Our lack of group differences for the Müller-Lyer same-different judgment task is also in line with previous research, as Happé [7] reported that autistic children were equally susceptible to the Müller-Lyer illusion as typically developing children, unlike for a range of other illusions in which they demonstrated reduced susceptibility.

What can we conclude from these apparently conflicting results? Do autistic children really perceive the Müller-Lyer illusion differently to typically developing children? It could be argued that Happé’s method (and that used in experiment 2) is too insensitive to reveal differences in the *extent* of illusion susceptibility between the groups, as it classifies children into those who do or do not experience the illusion. Indeed, it is clear from experiment 2 that the majority of children are susceptible to the Müller-Lyer illusion. However, it is particularly informative here that we found no group differences in the extent of bias in our novel 2-AFC method, which was specifically designed to reduce the influence of higher-level decisional strategies [29]. Thus, it is likely that any group differences in the method-of-adjustment Müller-Lyer task merely reflect differences in decisional criteria, rather than reflecting underlying differences in perception. It is interesting to note that Chouinard et al. [22] reported *reduced* susceptibility to the Müller-Lyer illusion in typical members of the population with high levels of autistic traits, as measured by the AQ. It seems that these results may not be generalizable to the autistic population, as no studies to date have reported reduced susceptibility to the Müller-Lyer illusion in those with a clinical diagnosis.

Our results on the Ebbinghaus illusion are very clear, as we found no group differences in susceptibility to the illusion in any method we used. These results fit within a complex pattern of results from previous studies, including both reports of reduced susceptibility (e.g., [7]), and no differences in susceptibility (e.g. [10, 16]) for autistic individuals. Such discrepant results may arise in part from the use of different methodologies. Yet, here we found no differences in susceptibility between autistic and typically developing children across three different methods, including a task based on Happé [7]. It should be noted, however, that our stimuli differed from those used by Happé and others. For example, our stimuli were presented in white on grey, whereas Happé’s stimuli were black and white, and the context circles in our Ebbinghaus stimuli did not touch, whereas they did in Happé’s stimuli. Stimulus differences such as these may be contributing factors in determining the extent to which autistic children are influenced by the Ebbinghaus illusion. A further difference is that we tested cognitively able autistic children (IQ > 70), whereas Happé tested autistic children with a lower range of IQ scores (verbal IQ range 40–92), although here we found no evidence of a correlation between bias and IQ in the Ebbinghaus tasks. It is possible that previous reports of reduced susceptibility to the Ebbinghaus task resulted from atypical decision strategies in autistic populations, on which sampling differences may have a particularly pronounced effect. Anecdotally, many of our participants reported ‘knowing’ the illusions from science books and TV shows, which may have substantially affected their responses in experiments 2 and 3. A large number of the children we tested did not answer the control question correctly in experiment 2 (*n* = 16 in the Ebbinghaus task). As the control stimuli were perceptually identical, such responses again point to a strong role for decisional biases.

Although we made extensive efforts to ensure that the samples tested in each experiment were of comparable age and non-verbal ability, it is a limitation of the current study that we were not able to test all experimental conditions within the same participants. The sample sizes used were relatively large for studies investigating susceptibility to visual illusions in an autistic population. Nevertheless, the exact sample size used varied between experiments and between groups of autistic and typically developing children. It is possible that the smaller samples were less sensitive to group differences than those with larger sample sizes. Indeed, our use of Bayesian statistics confirms the need for larger sample sizes to conclusively distinguish between the null and alternative hypotheses in certain conditions in experiments 2 and 3. Thus, future studies would benefit from collecting data from large samples for both the autistic and typically developing groups. Specifically, future research will need to confirm the key finding of increased bias to the Müller-Lyer illusion in the method-of-adjustment task in conjunction with similar levels of bias in the 2-AFC task, within the same sample of autistic participants.

Previous reports of reduced susceptibility to visual illusions have been linked to theories of autistic perception and cognition, such as weak central coherence [7] and reduced influence of top-down information [14, 26, 27]. The results of this study and other studies refute the suggestion that children on the autism spectrum have pervasively different responses to visual illusions compared to typically developing children. Indeed, the results from experiment 1 that measure perceptual bias suggest that autistic children process the context in the Ebbinghaus illusion and Müller-Lyer illusion similarly to typically developing children (cf. the weak central coherence theory [25]).

We may well expect distinct effects for different illusions. For example, autistic individuals may have reduced susceptibility to illusions that rely heavily on prior knowledge, such as the Shepard table illusion [14], despite not demonstrating reduced susceptibility to the Ebbinghaus illusion, which may result from lateral interactions in lower-level areas of the visual system such as V1 [49, 50]. A feasible hypothesis would be that we should only find atypical responses by autistic individuals to illusions that result from top-down processing. However, the state of existing research evidence does not yet allow us to make clear links with such theories, as previous reports of reduced susceptibility to illusions could be a result of atypical decisional strategies, rather than reflecting differences in perceptual processing. Adapting relatively bias-free methods to a range of different illusions will therefore be important in further investigating atypical visual perception in autism. One outstanding question is whether different illusions lead to differing levels of response bias. Indeed, the fact that we found significant group differences in performance in the method-of-adjustment Müller-Lyer task but not the Ebbinghaus task suggests that the Müller-Lyer illusion might be particularly sensitive to atypical decisional strategies—perhaps as a result of the illusion being stronger in general.

The methodological issues we highlight here are not restricted to studies of autism, and we stress the importance of designing studies that minimise decision biases whenever the focus is on underlying perceptual mechanisms. Our study demonstrates that the method developed by Morgan et al. [29], which is relatively free of cognitive bias, can be adapted successfully for children and clinical populations. Our use of a child-friendly ‘game’ context ensured that child participants were engaged with the task and sufficiently motivated to complete the trials. Future studies may benefit from employing a similar method in order to determine whether atypical responses to illusions in other clinical groups, such as schizophrenia (e.g. [51]), reflect real perceptual differences compared to neurotypical populations. The method could also be used to investigate perceptual development. While we found no evidence of age-related changes in bias in the current sample, it is possible that this would become evident in a larger sample of children across discrete age groups—allowing the possibility to confirm whether age-related changes in susceptibility to visual illusions [52–54] really reflect underlying changes in perceptual functioning. It is worth noting here that Káldy and Kovács [54] used a 2-AFC method with the intention of minimising bias when assessing the strength of the Ebbinghaus illusion in children. Yet, the 2-AFC method alone does not eliminate decisional bias, as observers can still guess in favour of one of the two options when they are unsure [29]. The combined use of a 2-AFC method with a roving pedestal, as demonstrated here, ensures that perceptual bias can be measured as purely as possible, in a wide range of populations.

## Conclusions

Using a new method to measure susceptibility to Ebbinghaus and Müller-Lyer illusions while minimising the contaminating effects of decisional biases, we found no evidence of differences in susceptibility between autistic and typically developing children. These results provide an important step in bridging behaviour with biological substrates, suggesting that group differences in susceptibility to illusions may emerge in higher-level decision-making rather than at the level of the percept.

## Additional files

Additional file 1: Results with no replacement of outliers. Means, standard deviations and *t*-test statistics for group differences in bias in the Ebbinghaus and Müller-Lyer tasks in experiment 1 and Müller-Lyer context-free judgments in experiment 3 when outliers were not replaced. (PDF 9 kb)

Additional file 2: Results of robustness checks for Bayesian analyses. Results of robustness checks for Bayesian independent sample *t* tests in experiments 1 and 3 and for the Bayesian contingency test in experiment 2. (PDF 92 kb)

## Abbreviations

2-AFC: two-alternative-forced-choice
ADOS: Autism Diagnostic Observation Schedule
AQ: Autism spectrum quotient
DSM: Diagnostic and Statistical Manual of Mental Disorders
FSIQ: Full-scale IQ
ICD-10: International Classification of Diseases-10
PIQ: Performance IQ
SCQ: Social Communication Questionnaire
VIQ: Verbal IQ
WASI-II: Wechsler Abbreviated Scales of Intelligence, 2nd Edition.

## Footnotes

1 Note that Ropar and Mitchell [10] previously considered the possibility that the performance of Happé’s [7] autistic individuals could have resulted from biases in verbal responses. However, the point we make here applies to responses made both verbally and non-verbally.

2 The pedestal sizes for the Ebbinghaus stimulus were taken from Morgan et al. [29]. Pilot testing revealed the need for a larger pedestal size for the Müller-Lyer illusion.
