# Supplementary material for: Susceptibility to Ebbinghaus and Müller-Lyer illusions in autistic children: a comparison of three different methods
Source: Mol Autism. 2017 Mar 23;8:16. doi: 10.1186/s13229-017-0127-y (PMC5364638; doi:10.1186/s13229-017-0127-y)
Supplement: Supplementary file 1 — Results with no replacement of outliers. Means, standard deviations and t-test statistics for group differences in bias in the Ebbinghaus and Müller-Lyer tasks in experiment 1 and Müller-Lyer context-free judgments in experiment 3 when outliers were not replaced. (PDF 9 kb) [file 13229_2017_127_MOESM1_ESM.pdf]

Additional File 1. Results with no replacement of outliers.

Experiment 1:

	Autistic M(SD)	Typically developing M(SD)	Results of independent-samples t-test
Ebbinghaus: Bias	39.62 (51.02)	54.44 (60.22)	*t*(60) = 1.04, *p* = .30 (bootstrapped CI [-44.89, 14.51], *p* = .34)
Müller-Lyer: Bias	77.70 (62.89)	70.58 (158.22)	*t*(78) = .25, *p* = .81 (bootstrapped CI [-43.20, 62.11], *p* = .80).

Experiment 3:

	Autistic M(SD)	Typically developing M(SD)	Results of independent-samples t-test
Müller-Lyer: Context-free	2.80 (6.44)	-.39 (3.75)	t(80) = 2.80, *p* = .006, *d* = 0.61
