# Supplementary material for: Susceptibility to Ebbinghaus and Müller-Lyer illusions in autistic children: a comparison of three different methods
Source: Mol Autism. 2017 Mar 23;8:16. doi: 10.1186/s13229-017-0127-y (PMC5364638; doi:10.1186/s13229-017-0127-y)
Supplement: Supplementary file 2 — Results of robustness checks for Bayesian analyses. Results of robustness checks for Bayesian independent sample t tests in experiments 1 and 3 and for the Bayesian contingency test in experiment 2. (PDF 92 kb) [file 13229_2017_127_MOESM2_ESM.pdf]

Additional File 2. Results of robustness checks for Bayesian analyses.

Results of robustness checks for Bayesian independent samples t-tests to assess group differences in bias in Ebbinghaus (A) and Müller-Lyer (B) tasks in Experiments 1 and 3, implemented using JASP Statistical Software (JASP Team, 2016).

<table>
<tr><td></td><td>Ebbinghaus</td><td>Müller-Lyer</td></tr>
<tr><td>Expt. 1</td><td>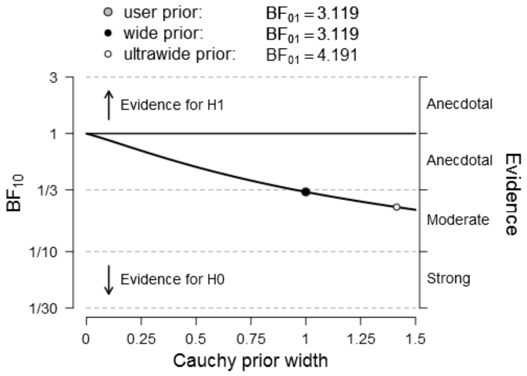
</td><td>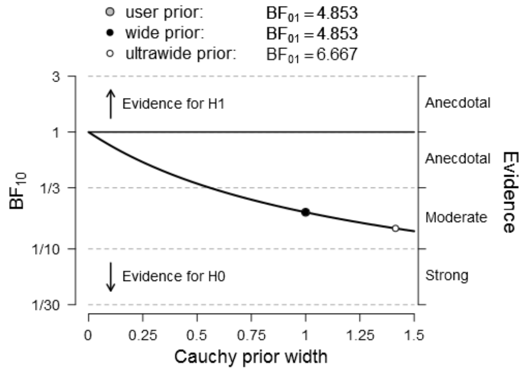
</td></tr>
<tr><td>Expt. 3</td><td>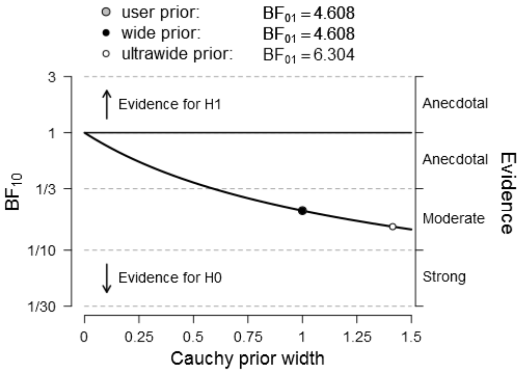
</td><td>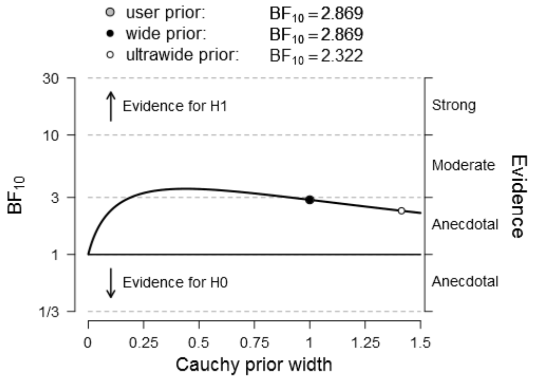
</td></tr>
</table>

Results of robustness check for Bayesian contingency test to assess independence between group membership and responses in the Ebbinghaus (A) and Müller-Lyer (B) task in Experiment 2. The Bayes Factor (BF_{10}) is plotted at a range of different prior concentrations.

	Ebbinghaus	Müller-Lyer
Expt. 2	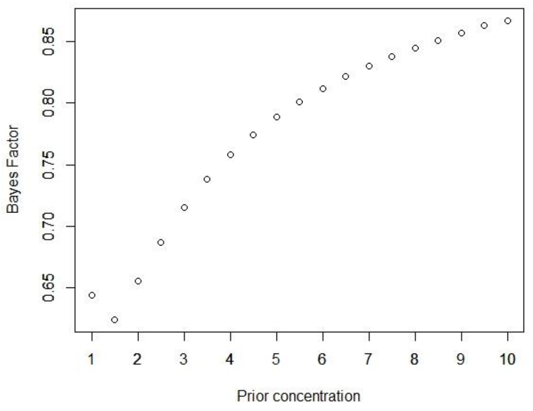	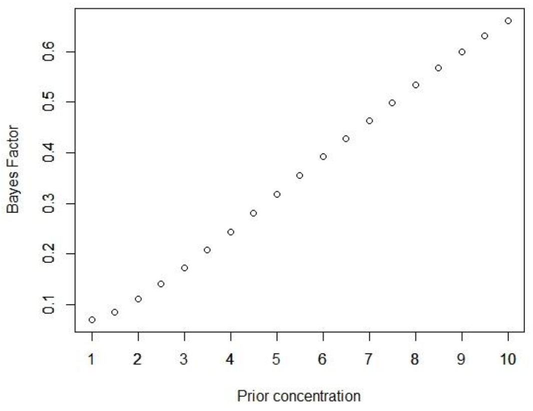
